# Developmental psychology: The expanding reach of emotions

**DOI:** 10.1038/s44271-023-00029-6

**Published:** 2023-10-20

**Authors:** Jennifer A. Bellingtier

**Affiliations:** Communications Psychology, Berlin, Germany

**Keywords:** Psychology, Development studies

## Abstract

For some individuals, daily changes in positive and negative emotions corresponds to fluctuations in overall life satisfaction. A new study in *Psychology and Aging* suggests that the expanding reach of negative emotions is greater for younger than older adults.

**Figure:**
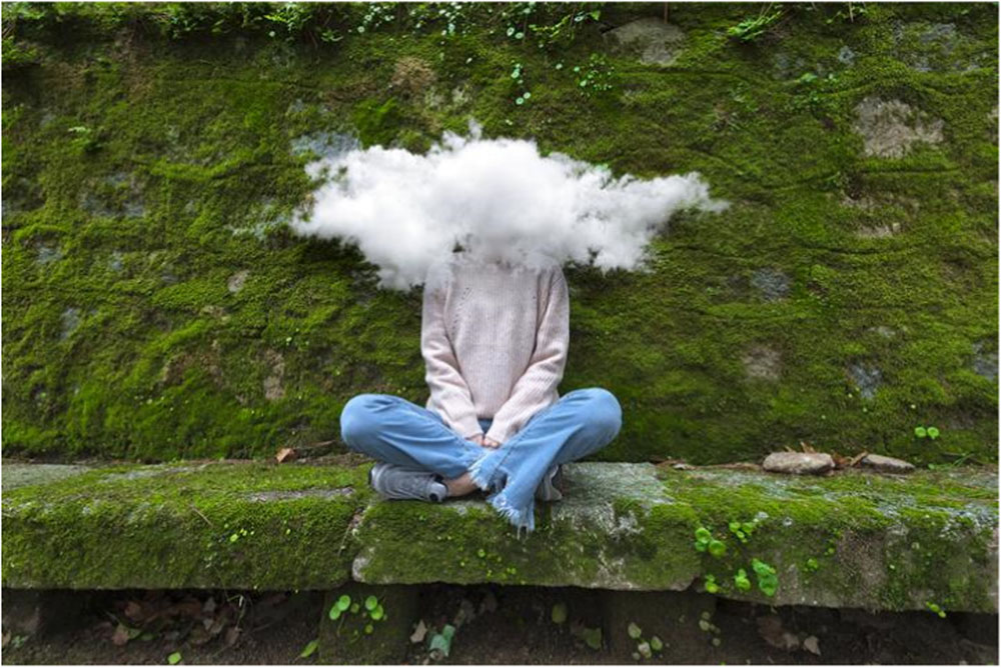
Francesco Carta for Getty Images

Emotions are typically transient feelings that fluctuate from day to day whereas evaluations of life-satisfaction have traditionally been thought to be relatively more stable. Emerging research suggests that for some individuals emotion fluctuations are also associated with daily changes in global life satisfaction, a phenomenon known as emotion globalizing.

Meaghan A. Barlow at Wilfrid Laurier University and colleagues investigated if the tendency to expand the reach of daily emotions was more pronounced in younger versus older adults^1^. Across two studies, they tracked positive and negative emotions either at the end of the day or following the day’s most stressful event as well as either global life satisfaction or satisfaction with the current day. They found that younger adults (ages 23–42 and 18–34), as compared to older adults (ages 51–79 and 64–95), were more likely to link their current-day negative emotions with more negative overall life evaluations. There were no age-related differences in the tendency to globalize positive emotions to either life or day satisfaction or negative emotions to day satisfaction. Interestingly, overall life satisfaction did not differ on average based on age, although average day satisfaction was higher for older adults.

These findings align with lifespan developmental theories suggesting that older age is associated with accrued wisdom and an improved ability to manage daily negative events. The ability to keep negative emotions from expanding may be one way older adults maintain life satisfaction into their later years.
